# Increased cortical activation upon painful stimulation in fibromyalgia syndrome

**DOI:** 10.1186/s12883-015-0472-4

**Published:** 2015-10-20

**Authors:** Nurcan Üçeyler, Julia Zeller, Susanne Kewenig, Sarah Kittel-Schneider, Andreas J. Fallgatter, Claudia Sommer

**Affiliations:** 1 Department of Neurology, University of Würzburg, Josef-Schneider-Str. 11, Würzburg, 97080 Germany; 2 Department of Psychiatry, University of Würzburg, Würzburg, Germany; 3 Department of Psychiatry, University of Tübingen, Tübingen, Germany

**Keywords:** Fibromyalgia syndrome, Near-infrared spectroscopy, Pain, Cortical activation, Depression

## Abstract

**Background:**

Fibromyalgia syndrome (FMS) is a chronic condition characterized by widespread pain and associated symptoms. We investigated cerebral activation in FMS patients by functional near-infrared spectroscopy (fNIRS).

**Methods:**

Two stimulation paradigms were applied: a) painful pressure stimulation at the dorsal forearm; b) verbal fluency test (VFT). We prospectively recruited 25 FMS patients, ten patients with unipolar major depression (MD) without pain, and 35 healthy controls. All patients underwent neurological examination and all subjects were investigated with questionnaires (pain, depression, FMS, empathy).

**Results:**

FMS patients had lower pressure pain thresholds than patients with MD and controls (p < 0.001) and reported higher pain intensity (p < 0.001). Upon unilateral pressure pain stimulation fNIRS recordings revealed increased bilateral cortical activation in FMS patients compared to controls (p < 0.05). FMS patients also displayed a stronger contralateral activity over the dorsolateral prefrontal cortex in direct comparison to patients with MD (p < 0.05). While all three groups performed equally well in the VFT, a frontal deficit in cortical activation was only found in patients with depression (p < 0.05). Performance and cortical activation correlated negatively in FMS patients (p < 0.05) and positively in patients with MD (p < 0.05).

**Conclusion:**

Our data give further evidence for altered central nervous processing in patients with FMS and the distinction between FMS and MD.

**Trial registration:**

ISRCTN registry ID ISRCTN15015327 (24.09.2015).

**Electronic supplementary material:**

The online version of this article (doi:10.1186/s12883-015-0472-4) contains supplementary material, which is available to authorized users.

## Background

Fibromyalgia syndrome (FMS) is a chronic pain condition with a clinically well-defined presentation [1], but of unknown etiology. FMS can be diagnosed according to published criteria and involves chronic widespread pain with additional symptoms like sleep disturbance or fatigue [2, 3]; patients also often complain of cognitive impairment. It is assumed that altered processing of nociceptive stimuli in the central nervous system (CNS) is involved in FMS pain. Patients with FMS have been investigated with different radiological and nuclear medicine methods for clarification of possible morphological or functional CNS alterations compared to healthy controls [4–7]. The current concept on the role of the CNS in FMS pain is mainly based on the assumption of increased central sensitization [8–10], lack of central inhibition [11, 12], and lack of central analgesic receptors [13], while peripheral input is also pathologically altered [14–16].

Functional near-infrared spectroscopy (fNIRS) is an easily applicable imaging technique that has no known side effects. fNIRS allows the investigation of cerebral activity as a function of cortical concentration changes of oxygenated and deoxygenated hemoglobin. Based on data from functional magnetic resonance imaging (fMRI) studies [17], we hypothesized that pain associated cortical activation in FMS patients is stronger and has a wider spatial distribution compared to controls that can be detected with fNIRS. To test this hypothesis we performed fNIRS under painful stimulation in groups of patients with FMS, unipolar major depression (MD) without pain, and healthy controls. Since cognitive impairment is frequently reported by FMS patients and supporting findings have been reported [18] we additionally performed fNIRS under cognitive stimulation.

## Methods

### Subjects

Our study was approved by the Würzburg Medical School Ethics Committee and written informed consent was obtained from all study subjects prior to inclusion. We included 25 FMS patients (23 women and two men) diagnosed according to the 1990 criteria of the American College of Rheumatology (ACR) [3]. Patients were recruited from all over Germany between 2007 and 2012 (Additional file 2: Figure S1). Inclusion criteria were: male and female patients ≥18 years; other possible differential diagnoses excluded (e.g. rheumatologic, orthopedic); no clinically relevant psychiatric disorder (examined by systematic psychiatric interview); willingness to participate in all tests during the study. The following exclusion criteria were applied: pain of other origin (e.g. rheumatoid arthritis; post-surgery pain); current or prior cerebral disease (e.g. stroke, cerebral hemorrhage, head trauma). The median age of the FMS patients was 59 years (range 50–70 years). All patients were examined at the Department of Neurology at the University Hospital of Würzburg, Germany.

We also investigated a group of ten patients (nine women, one man; n = 7: in-patients, n = 3: out-patients) with unipolar major depression (MD) without pain history as disease controls. We included this group to control for possible confounding effects of depression on the study results, because depressive symptoms are frequently observed in FMS patients. Patients were recruited at the Department of Psychiatry at the University Hospital of Würzburg between 2010 and 2011. The diagnosis was made by two trained psychiatrists according to the ICD-10 DCR (Diagnostic Criteria for Research; http://www.who.int/classifications/icd/en/GRNBOOK.pdf). All patients suffered from recurrent depressive episodes. The median age of the patients with MD was 50 years (range 39–75). Patients were not included in the following cases: pain of any source; psychiatric comorbidities (bipolar-affective disorder, schizophrenia, anxiety disorder, obsessive-compulsive disorder, eating disorder or substance abuse); current or prior cerebral diseases (e.g. stroke, cerebral hemorrhage, head trauma).

The control group consisted of 35 healthy volunteers (31 women, 4 men). Subjects were interviewed to exclude comorbidities like pain, depression and other psychiatric disorders, or cerebral disorders. The control group was matched with the FMS patient group as for age, gender, and educational background. The median age was 59 years (range 29–70). Data on the peripheral nervous system of our patients have been published recently [15]. All study participants were right-handed. Additional file 1: Table S1 gives further details on the patient groups.

### Clinical examination and questionnaire assessment

All patients were investigated neurologically by the same investigator and the diagnosis of FMS was confirmed. For pain characterization we applied the German version of the Graded Chronic Pain Scale (GCPS) [19]. From the GCPS we used the mean value of the three pain intensity items as an indicator of pain severity, and the mean value of the three items rating pain interference with social, occupational, and recreational activities as a disability score. To assess depressive symptoms the validated German version of the Beck Depression Inventory II (BDI) was applied scanning the last two weeks [20]. The calculated scores translate to >14 mild, >20 medium, >29 severe depression. The German version of the Fibromyalgia Impact Questionnaire (FIQ) was used to determine FMS associated symptoms, daily impairment, and overall well-being in the last week [21]. The FIQ was used instead of the revised FIQ, which was published after study initiation. A maximum FIQ score of 80 can be reached. All study participants were also assessed with the German “Saarbrücker Persönlichkeitsfragebogen” (SPF) (http://psydok.sulb.uni-saarland.de/volltexte/2009/2363/) that is based on the Interpersonal Reactivity Index (IRI) [22]. This multidimensional questionnaire tests for different components of empathy and contains one cognitive (perspective taking) and three emotional dimensions (fantasy, personal distress, empathic concern).

### Functional NIRS recordings (fNIRS)

#### Data acquisition

We investigated cortical activation of the study participants with fNIRS during two tasks:

1. Muscular pressure pain: fNIRS measurements were performed during the application of painful pressure on the muscle bulk of the finger extensors of the dominant hand side (which was the right side in all cases) using a calibrated algesiometer (Wagner Instruments, USA; Additional file 3: Figure S2). For this stimulation we first determined the individual pressure pain threshold by applying increasing pressure with the algesiometer until the study participant reported pain. Pain intensity at the individual pain threshold was rated on a numeric rating scale (NRS) with zero (no pain at all) to 100 (worst pain imaginable). Having identified individual pressure pain thresholds, we could then calibrate pressure stimulation to apply either painful (5 N above the threshold) or non-painful (5 N below the threshold) pressure. A total of 20 painful and 20 painless stimuli were applied in a randomized order. For computer aided randomization we used Presentation Software (Neurobehavioral Systems Incorporation, USA) so that the stimulus to be applied (painful or painless) was presented to the investigator on a monitor. The stimulation conditions were as follows: pressure application for two seconds; pause for ten seconds between two stimuli to allow hemodynamic response to return to baseline; no two stimulations at the same location. Additionally, we investigated ten randomly chosen FMS patients and matched healthy controls with a control condition: FMS patients and healthy controls were stimulated with a pressure intensity just at the median pain threshold of FMS patients, which was rated non-painful by healthy controls. Otherwise, the same procedure as described above was used. This experiment was included to investigate the extent of cortical activation when both study groups were stimulated with the same pressure intensity which was painful for FMS patients but painless for healthy controls.
2. Verbal fluency test (VFT): The VFT paradigm consisted of three conditions. Subjects were asked to produce as many different nouns as possible a) starting with a certain letter (A, F, and S), or b) belonging to the same category (animals, fruits, and flowers) or c) to name the days of the week as a control condition. Each condition lasted for 30 sec followed by 30 sec rest. Subjects worked on nine blocks in total (3 × letters, 3 × categories, 3 × week days). The VFT paradigm was used for two reasons: i) it reliably activates the frontal cortex and was thus an internal control, ii) FMS patients often complain of difficulties in word finding [23].

#### Data assessment

Concerning fNIRS data we assessed the difference in O_2_Hb levels comparing recordings between baseline and post-stimulation periods. Previous studies showed that O_2_Hb is more sensitive to regional changes in cerebral blood flow than HHb [24, 25], and that cranial and cutaneous circulation does not change main fNIRS results [26]. During fNIRS recordings a moving average of 5 sec and a high pass filter were applied using the implemented ETG-4000 software. After data export fNIRS records for local pressure pain stimulation were analyzed with a model based approach for event related study designs [27]. First, data underwent a low pass filter and a discrete cosine filter. In the next step, a hemodynamic response function (hrf) was constructed for every stimulus. With a peak time of 6.5 sec and a Gaussian shape it represented the expected hemodynamic response after every stimulus and served as a predictor in the next step of the analysis. Using a general linear model as an established model in fNIRS and also in fMRI studies [28] beta weights were estimated for each stimulus based on the modeled hrf and averaged for every condition and every channel in each subject. Data of VFT (block design) were averaged for the nine task periods (30 sec) with an onset delay of 3 sec, a trial length of 27 sec, and a baseline starting 3 sec before the onset of the task for each channel in every subject. Afterwards data were averaged for the three conditions. The onset delay was used to make sure to process only fNIRS data during the VFT task as it took subjects sometimes one or two seconds to start the task after the prompt. Both tasks activate the DLPFC, which is the interface between the pain neuromatrix and the neuronal network of cognitive functions [29]. Task 1) and 2) were applied in randomized order. Study subjects were seated in a quiet and dark room in front of a monitor and a keyboard; they were instructed to relax, avoid movements, and close their eyes. Cerebral activation leads to an initial drop in local oxygenated haemoglobin (O_2_Hb) levels followed by a compensatory hyperperfusion and oxygenation of the active brain area [30]. This secondary rise in local oxygen levels is recorded via fNIRS as a function of increased brain activity. We used a continuous wave NIRS device (ETG-4000 Optical Topography System; Hitachi Medical Corporation, Tokyo, Japan) that emits light at wave lengths of 695 ± 20 nm and 830 ± 20 nm and has a temporal resolution of 10 Hz. Two 3x5 probe sets of optodes (8 emittors, 7 detectors; Additional file 4: Figure S3) forming 22 channels were placed bilaterally over the dorsolateral prefrontal cortex (DLPFC) and the primary and secondary somatosensory cortex. For all channels, the emitter-detector separation was 3 cm, which translates to a spatial resolution of 0.5–2 cm depth [31, 32]. The second-last optode of each probe set was placed over the electrode positions T3 and T4 (detectors) respectively according to the international 10–20 system (Additional file 4: FigureS3). The anatomical correlates were determined according to Okamoto et al. [33].

#### Number connection test

To differentiate between potential executive dysfunction and generalized cognitive impairment particularly in low-performing patients in the VFT, we applied the German “Zahlenverbindungstest”, which is a simple number connection test (NCT) [34]. The NCT is a simple tool for testing cognitive speed and performance that is frequently reported to be reduced in FMS patients. Each study participant was presented four sheets with randomly written numbers from zero to 90 and was asked to connect these numbers in ascending order by drawing a line between. The time needed for completing the task was determined.

#### Statistical analysis

We used MATLAB 7 (The MathWorks, Ismaning, Germany) and the IBM PASW Statistics 19.0 software (Munich, Germany) for statistical analysis. The Kruskal-Wallis test was applied for data comparison of non-normally distributed data; these data were illustrated as boxplots giving the median, the upper 75 % and lower 25 % percentiles and the minimum and maximum values. For correlation analyses in normally distributed data we used the bivariate Pearson correlation. P < 0.05 was assumed significant. For statistical analysis of fNIRS data 2 × 3 ANOVAs for repeated measures were conducted for the pressure pain stimulation comprising the factors “condition” (painful stimulation and non-painful stimulation) and “group” (FMS patients, patients with depression, and healthy controls). To analyze the VFT paradigm 3 × 3 ANOVAs were calculated containing the factors “condition” (letter version, category version, and weekdays) and “group” (FMS patients, patients with MD, and healthy controls). Blocks within each condition were averaged for each channel. ANOVAs were conducted separately for each channel. Post-hoc comparisons used t-tests for dependent and independent samples as appropriate. The modified Bonferroni adjustment according to Dubey and Armitage-Parmar was used to correct for multiple testing [35]. This method considers the high spatial correlation between NIRS channels and has been used in numerous publications (e.g. [36, 37]). Corrected alpha-levels and critical t-values for each comparison are listed in every legend as well as the spatial correlation (Pearson).

## Results

### Clinical findings

Neurological examination was normal in all patients. There was no significant difference in demographics between the groups.

Pain intensity and impairment due to pain was higher in FMS patients compared to patients with depression and to healthy controls (Additional file 5: Figure S4A-C, p < 0.001 each), while FMS patients and patients with MD did not differ in depressive symptoms (Additional file 5: Figure S4D). FMS patients reached higher scores in the FIQ compared to patients with MD (p < 0.01) and to controls (p < 0.001; Additional file 5: Figure S4E). In the SPF patients with FMS showed higher scores for personal distress (p < 0.01) and empathic concern (p < 0.05) compared to healthy controls.

### Patients with FMS have lower pressure pain thresholds and report on higher pain intensities

FMS patients had the lowest median pressure pain thresholds (16, 13–27 N) compared to patients with depression (35, 25–55 N) and healthy controls (38, 25–60 N, p < 0.001 each). Interestingly, subjective median pain intensity at the threshold pressure was higher in FMS patients (72, 52–88 NRS) than in controls (54, 34–85, p < 0.05). The pain threshold and the beta weight difference between the painful and the non-painful stimulation during the fNIRS measurement did not correlate in any of the groups (r < ±0.344, p > 0.05).

### FMS patients show bilateral cerebral activation upon unilateral painful stimulation

**Fig. 1:**
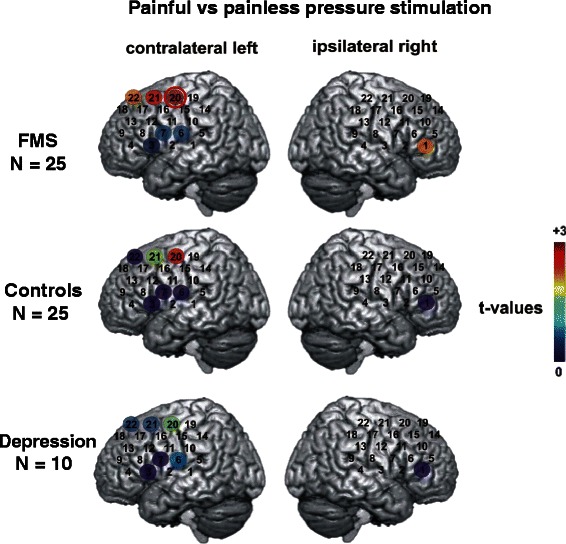
Cerebral activity in fNIRS recordings after painful versus painless pressure stimulation. Coloured circles present the results of t-tests comparing the beta weights between two conditions (painful versus painless stimulation) for each of the three study groups for significant channels. Each circle shows the t-value of the comparison in each channel (indicated by the colour bar, where dark red represents the largest t-value [+3] and dark blue the smallest t-value [0]). Data from the contralateral hemisphere are presented in the left column while those from the ipsilateral hemisphere are presented in the right column. The channel marked with a red circle withstands the DAP correction for multiple testing (left side r_59_ = 0.441, critical t-value (two-sided) = 2.69, α = 0.009; right side r_59_ = 0.455, critical t-value (two-sided) =2.67, α = 0.01)

**Fig. 2:**
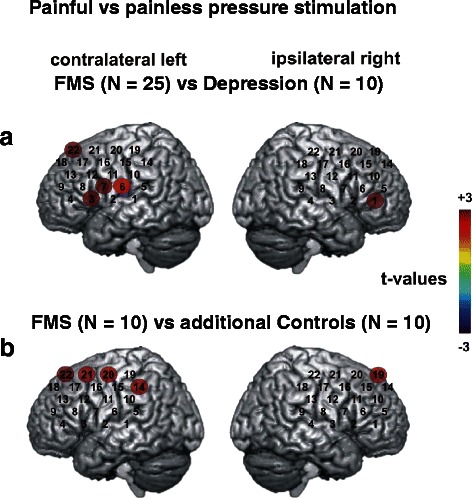
Cerebral activity in fNIRS recordings after painful pressure stimulation comparing FMS patients with patients with depression and with healthy controls. **a** Coloured circles present the results of DAP corrected post-hoc t-tests comparing the beta weights between two conditions (painful versus painless stimulation) for the comparisons between patients with fibromyalgia syndrome (FMS) and depression (left side r_59_ = .441, critical t-value (two-sided) =2.69, α = 0.009; right side r_59_ = 0.455, critical t-value (two-sided) = 2.67, α = 0.01). Each circle shows the t-value of the respective comparison in each channel (indicated by the colour bar, where dark red represents the largest t-value [+3] and dark blue the smallest t-value [−3]). Data from the contralateral hemisphere are presented on the left side while those from the ipsilateral hemisphere are presented on the right side. Painful pressure stimulation leads to a much stronger cerebral activation in FMS patients than in patients with depression. **b** Coloured circles present the results of DAP corrected post-hoc t-tests comparing the beta weights between two conditions (stimulation with a pressure intensity just at the pain threshold of FMS patients, i.e. painless for healthy controls) for the comparisons between patients with fibromyalgia syndrome (FMS) and healthy controls for significant channels (left side r_59_ = 0.441, critical t-value (two-sided) = 2.69, α = 0.009; right side r_59_ = 0.455, critical t-value (two-sided) = 2.67, α = 0.01). Each circle shows the t-value of the respective comparison in each channel (indicated by the colour bar, where dark red represents the largest t-value [+3] and dark blue the smallest t-value [−3]). Data from the contralateral hemisphere are presented on the left side while those from the ipsilateral hemisphere are presented on the right side. The stimulation leads to a bilateral cerebral activation only in FMS patients

Assessment of fNIRS data for the three groups (FMS patients, MD patients and healthy controls) revealed a significant main effect “condition” for channels 3, 6, 7, 20, and 21 of the probe set placed over the left hemisphere (10.5 > F_(1.57)_ >4.24, p < 0.05) and a significant interaction “group x condition” in channels 6, 7, and 22 on the left and in channel 1 on the right hemisphere probe set (5.44 > F_(2.57)_ >3.24, p < 0.05). Regardless of the group all subjects showed a stronger increase of [O_2_Hb] in the control condition in channels 3, 6, and 7 and a stronger increase during the pain condition in channels 20 and 21. Post-hoc comparisons between conditions for the seven significant channels showed a stronger increase in [O_2_Hb] for the painful stimulation of FMS patients in channels 20, 21, and 22 over the left and in channel 1 over the right hemisphere (t_(24)_ > 2.0, p < 0.05). Channels with significant differences were located over the DLPFC and primary and secondary somatosensory cortices (Fig. 1). Healthy controls showed greater activity only in channel 20 on the left hemisphere. Patients with MD displayed no differences in activation between the conditions. After correcting for multiple testing only channel 20 in FMS patients remained significant (Fig. 1). The direct comparison of the cerebral activation in patients with FMS and patients with MD in the seven significant channels revealed that FMS patients had a higher activation in four channels over the left (3, 6, 7, and 22) and in channel one over the right hemisphere (t_(33)_ > 2.71, p < 0.01; Fig. 2A). When comparing FMS patients and controls directly the group differences did not reach statistical significance.

### At-threshold pain stimulation induces cerebral activation only in FMS patients

As described above an additional control condition was applied to ten randomly chosen FMS patients and ten matched controls. Subjects were stimulated with a pressure intensity of 15 N that was the median value of the pain threshold of FMS patients but painless for healthy controls. Under this condition an ANOVA displayed a main effect of “group” in four channels over the left hemisphere (1, 5, 6, 7) and in channel 6 over the right hemisphere (4.7 < F_(1,18)_ < 8.7, p < 0.05) and an interaction “group × condition” in ten channels on the left (9, 13, 14, 16, 17, 18, 19, 20, 21 and 22) and three channels on the right side of the probe set (19, 20 and 21; 4.7 < F_(1,18)_ < 9.0, p < 0.05). Regardless of the nature of the stimulus FMS patients showed less changes in [O_2_Hb] in five channels (1, 5, 6, 7 on the left and 6 on the right side). Corrected post hoc t-tests for the interaction “group × condition” revealed a stronger increase in [O_2_Hb] for the pain stimulus in contrast to the painless stimulus for FMS patients then in healthy controls in channels 14, 20, 21, and 22 on the left side and in channel 19 on the right side of the probe set (t_(33)_ > 2.7, p < 0.01; Fig. 2B). FMS patients thus showed a clearly more pronounced cerebral activation in response to the painful stimulus also in channels covering the DLPFC.

### Cognitive performance is not different between FMS patients and controls, while prefrontal activation is distinct between patients with FMS and depression

**Fig. 3:**
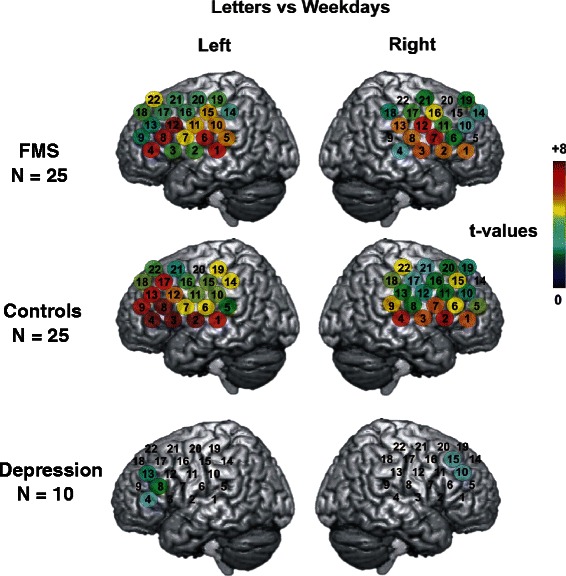
Cerebral activity in fNIRS recordings during the verbal fluency test. Coloured circles present the results of DAP corrected t-tests comparing the beta weights between two conditions for each of the three study groups during the verbal fluency test (VFT); left side r_59_ = 0.467, critical t-value (two-sided) = 2.66, α = 0.01; right side r_59_ = 0.48, critical t-value (two-sided) = 2.64, α = 0.01). Each circle shows the t-value of the comparison in each channel (indicated by the colour bar, where dark red represents the largest t-value [+8] and dark blue the smallest t-value [0]). Data from the contralateral hemisphere are presented in the left column while those from the ipsilateral hemisphere are presented in the right column. Patients with FMS did not differ in cerebral activation, while patients with depression showed a reduction of frontal cerebral activation

Patients with FMS performed as well as patients with depression or healthy controls in the VFT (1.61 < F_(2.59)_ < 2.21, 0.12 < p < 0.22) and in the NCT. For the fNIRS data ANOVAs showed a significant main effect “condition” for all channels (F_(2.114)_ >5.0, p < 0.01) and an interaction “groups × condition” for nine channels on the left and three channels on the right side of the probe set (F_(4.114)_ > 2.5, p < 0.05). All subjects displayed a stronger increase in [O_2_Hb] during the letter condition as compared to the weekday condition (t_(59)_ > 3.75, p < 0.001). The same was observed for the category versus the weekday condition (t_(59)_ 2.76, p < 0.01) except for channel 9 on the right side where no significant difference could be found (t_(59)_ = 0.39, p = 0.70). For the greater part of the probe set the increase in oxygenated hemoglobin during the letter condition exceeded the category condition (t_(59)_ > 2.15, p < 0.05). Only channels 16, 18, 19, 20, and 21 on the right and channels 14, 19, 20, 21, and 22 on the left side showed no difference between the conditions (t_(59)_ < 1.93, p > 0.05). While fNIRS measurements showed no difference in cortical activation during the VFT between patients with FMS and healthy controls, patients with MD displayed a frontal deficit of cortical activation. They showed a smaller increase in [O_2_Hb] (corrected for the weekday task) as compared to FMS patients during the letter condition (left side channels 6, 7, 11, 12, 15, 16, and 22; t_(33)_ >2.66, p < 0.01; right side channels 3 and 13; t_(33)_ > 2.64, p < 0.01). During the category task only channel 22 on the left side showed a significant difference between patients with MD and with FMS (t_(33)_ = 2.93, p < 0.01). The same was observed when comparing MD patients and controls (letter condition: left side channels 2 ,3, 6, 7, 8, 11, 12, 14, 15, and 16; t_(33)_ > 2.66, p < 0.01; right side channels 2, 3, 4, and 18; t_(33)_ > 2.64, p < 0.01; category condition: left side channels 12, 15, and 22; t_(33)_ > 2.75, p < 0.01; right side channel 22; t_(33)_ = 3.18, p < 0.01). The frontal deficit displayed by MD patients in comparison to healthy controls during the VFT was described previously [38] (Fig. 3).

### FMS patients with lower cortical activation perform better in the VFT than those with high cortical activation

For the category task of the VFT no correlation was found between performance and cortical activity in any of the three study groups. In the letter task however, FMS patients showed a negative correlation between performance and cortical activity as measured with fNIRS. FMS patients producing a higher number of correct words displayed lower task related cortical activation (Additional file 5: Figure S4). Correlations reached significance in channels 10, 14, 15, 20, and 21 over the left and in channels 11, 13, 17, 18, 19, and 21 over the right hemisphere (r > −0.40, p < 0.05; Additional file 5: Figure S4). In contrast, patients with MD showed a positive correlation between the number of correct words beginning with a certain letter and oxygenation changes: cortical activity was increased in channels 1, 8, and 10 over the left and in channels 1, 2, 7, and 11 over the right hemisphere (r > 0.65, p < 0.05). Patients with MD producing a higher number of correct answers showed greater cortical activation in temporo-frontal areas (Additional file 6: Figure S6). In controls no such correlation was found and also no correlation was found for the weekday task.

## Discussion

This is the first report on bilateral cerebral activation following unilateral painful stimulation in patients with FMS as measured by fNIRS. Our study adds to the growing evidence of an augmented cerebral activation upon painful stimulation as one contributor to pain in FMS. Additionally, clear differences in cortical activation during a cognitive task could be observed between patients suffering from FMS and MD.

Our data are in line with the results of fMRI studies in FMS patients where painful stimulation with identical stimulus intensities led to an augmented cerebral activation of pain-related brain areas of FMS patients compared to healthy controls [5, 17, 39]. The investigation of the DLPFC as an important part of the descending pain modulatory system is of major interest [40]. Mere anticipation of pain already elicited greater DLPFC activation in FMS patients as compared to healthy controls [41]. In a recent study using 2D-chemical shift imaging MR-spectroscopy sequences, the choline/creatine variability in the right DLPFC was different between FMS patients and healthy controls and correlated with pain in FMS patients [7]. Also, we confirm the augmented cerebral activation in FMS patients at pressure levels that are not painful for controls [17]. Similarly, FMS patients displayed a bilateral and stronger cerebral activation upon thermal stimulation while controls only showed a contralateral activation [42]. Our data also confirm previous findings that patients with FMS have lower pressure pain thresholds compared to controls and rate higher pain intensities upon identical stimulus intensity.

fNIRS is an imaging technique that has only been applied in few pain studies so far. Since no side effects are known NIRS has mostly been used in newborns and children (e.g. [43]. In one study the temporal and spatial characteristics of somatosensory cortex activation was investigated; NIRS differentiated between mechanically painful and non-painful stimuli [44]. In another study a correlation was found between fNIRS signals and patients’ subjective acute pain levels [45]. NIRS was also used to visualize pain during painful procedures like in cardiac surgery [46] or arthroscopic shoulder surgery [47] and in migraineurs [48]. In a recent study fNIRS was applied in rats to study the changes in blood hemoglobin levels during painful and non-painful stimulation [49].

Our study contributes to the ongoing debate on whether FMS is an independent entity or a variant of depression. Here we provide further evidence for a distinct pathophysiology of FMS compared to MD. We show that FMS patients differ in their cortical activity from patients with MD but without pain by a) stronger and bilateral cortical activation upon painful stimulation, b) normal cortical activation during executive functions (VFT), and c) a higher cortical activation during the letter task of the VFT correlating with low performance. The prefrontal cortex plays a crucial role in executive functioning [50]. Similar to patients with depression, FMS patients usually perform worse on tests targeting working memory, executive control, and attention [51, 52]. Distinct from patients with FMS, patients suffering from depression showed a lower cortical activation during the VFT which lines up with previous findings [38]. The VFT is a well-established paradigm for the investigation of executive frontal brain functioning [53] and reliably elicits DLPFC activation [54]. As shown before we did not find a difference in the VFT when comparing the performance of FMS patients with age-matched healthy controls [55]. We also did not find indications of cognitive impairment or reduced mental performance although patients subjectively report on such symptoms as previously described [56]. However, one striking finding of our study is that increased cortical activation in FMS patients correlated negatively with performance in the letter task of the VFT, while in patients with MD higher cortical activity correlated with better performance. Together with the augmented cortical activation upon low grade pressure pain stimulation observed in FMS patients this phenomenon might be due to a predisposition for over-activation of the nervous system.

Our study has several limitations. Our group of patients with FMS is relatively small, which might be the reason for the lack of correlation between pain intensities or thresholds compared to cortical activity as measured by fNIRS. Also, the group of patients with MD is small and we have not investigated a group of patients with concomitant depression and pain. This was due to the low mental and physical endurance of these severely affected patients who disagreed to participate in such a tedious study with several different tasks. Furthermore, NIRS only reaches superficial and selected brain areas. However, in accordance with previous data [54, 57], our results show that reliable and reproducible fNIRS recordings can be obtained with the paradigms used and the findings line up well with previous data of fMRI studies. Investigations so far revealed no evidence for a substantial influence of fNIRS recordings by non-neuronal confounders like extra-cerebral circulation or muscle activity [26].

The advantages of NIRS as compared to MRI outweigh the limitations for studies examining FMS patients: NIRS is easy to perform and convenient for the patient; NIRS has almost no exclusion criteria and no methodological limitations such as ferromagnetic objects or narrow scanners that hinder stimulus application. Furthermore, fNIRS has a high ecological validity and allows an investigation in sitting position, without head fixation, scanner noise, and anxiety-inducing surrounding. So far, fNIRS as well as fMRI do not allow individual data analysis; both methods are limited to group analyses. Therefore fNIRS also is not suitable to visualize pain in the clinical routine of individual patients. Thus, new approaches are needed to decipher the underlying mechanisms of pain particularly in complex conditions like FMS [58]. The major contribution of our explorative study is that fNIRS as an easy to apply new imaging technique without side effects is suitable to investigate pain-associated cortical activity. Furthermore, FMS patients show a cortical activation pattern upon painful stimulation that is distinct from healthy controls and especially from patients with depression. This strengthens the notion that FMS is an independent entity rather than being a mere variant of depression.

## Conclusion

The major contribution of our explorative study is that fNIRS as an easy to apply new imaging technique without side effects is suitable to investigate pain-associated cortical activity. Furthermore, FMS patients show a cortical activation pattern upon painful stimulation that is distinct from healthy controls and especially from patients with depression. This strengthens the notion that FMS is an independent entity rather than being a mere variant of depression.

## Additional files

Additional file 1: Table S1. Characteristics of patients with fibromyalgia syndrome (FMS) and monopolar depression. (DOC 77 kb)
Additional file 2: Figure S1. Flow-chart of patient enrolment. The flow-chart shows the process of eligibility testing of the FMS patients before enrolment. (PPT 72 kb)
Additional file 3: Figure S2. Pressure pain stimulation. A technician of our group demonstrates the application of pressure on the muscle bulk of the finger extensors using a calibrated algesiometer (Wagner Instruments, USA). (PPT 2368 kb)
Additional file 4: Figure S3. Positioning of probe sets. The positions of the emitters (red dots) and the detectors (blue dots) are illustrated over the left and right hemisphere. The channels between the optodes are consecutively numbered. T3, T4, Fp1 and Fp2 are standard electroencephalography points that mark the positions of the detectors between channel 1 and 2 on the left side and channel 3 and 4 on the right side. (PPT 208 kb)
Additional file 5: Figure S4. Results of questionnaires for pain, depression, and FMS symptoms. The boxplots give the results of the questionnaire assessment of the study participants. The median, and the first and third quartile are illustrated. (A) Patients with FMS had a higher median pain intensity in the last four weeks as measured by the Graded Chronic Pain Scale (GCPS) compared to patients with depression and to controls. (B) Also impairment of daily life due to pain in the last four weeks was greater in the FMS group compared to the other groups. (C) When asked for the current pain intensity FMS patients reported higher scores on a numerical rating scale (zero: no pain; ten: worst imaginable pain) then patients with depression or healthy controls. (D) In the Beck Depression Inventory (BDI) depressive symptoms were present in the FMS group and also in the group with unipolar depression compared to controls. (E) The Fibromyalgia Impact Questionnaire (FIQ) revealed higher scores for the FMS group compared to the depression group and the controls. *p < 0.05, **p < 0.01, ***p < 0.001. (PPT 107 kb)
Additional file 6: Figure S5. Correlation of performance in the verbal fluency test and cortical activation. Correlation between the task related activation in channel 10 over the left hemisphere and the number of correct answers during the letter version of the verbal fluency test (VFT) for patients with FMS and depression (p < 0.05). (PPT 323 kb)

## Abbreviations

CNS: Central nervous system
fMRI: Functional magnetic resonance imaging
FMS: Fibromyalgia syndrome
fNIRS: Functional near-infrared-spectroscopy
MD: Monopolar depression
VFT: Verbal fluency test
