# Supplementary material for: Increased cortical activation upon painful stimulation in fibromyalgia syndrome
Source: BMC Neurol. 2015 Oct 20;15:210. doi: 10.1186/s12883-015-0472-4 (PMC4618366; doi:10.1186/s12883-015-0472-4)
Supplement: Additional file 1: Table S1. — Characteristics of patients with fibromyalgia syndrome (FMS) and monopolar depression. (DOC 77 kb) [file 12883_2015_472_MOESM1_ESM.doc]

**Additional file 1: Table S1:** Characteristics of patients with fibromyalgia syndrome (FMS) and monopolar depression.

| **Patient, gender**  **(M: male,**  **F: female)** | **Age (yrs)** | **Disease duration**  **(yrs)** | **Profession** | **Current analgesic and antidepressive medication** |
| --- | --- | --- | --- | --- |
| **FMS** |  |  |  |  |
| 1, M | 53 | 20 | Technician | None |
| 2, F | 62 | 25 | Industrial sales representative (retired) | None |
| 3, F | 67 | 21 | Office worker (retired) | Ibuprofen on demand |
| 4, F | 59 | 15 | Industrial sales representative (retired) | None |
| 5, F | 59 | 28 | Office worker | None |
| 6, F | 63 | 26 | Dental technician | None |
| 7, F | 54 | 12 | Doctor`s assistant | Piroxicam on demand |
| 8, F | 60 | 7 | Lawyer | Ibuprofen on demand |
| 9, F | 62 | 45 | Staff executive (retired) | Paracetamol on demand, homoeopathic substances |
| 10, F | 61 | 17 | Tailor | Amitriptyline |
| 11, F | 52 | 27 | Secretary | Amitriptyline, tramadol |
| 12, F | 56 | 44 | Office worker | None |
| 13, F | 54 | 10 | Highschool teacher | None |
| 14, F | 68 | 21 | Highschool teacher | None |
| 15, F | 57 | 20 | Office worker (retired) | None |
| 16, F | 65 | 45 | Assistant tax consultant (retired) | None |
| 17, F | 54 | 36 | Doctor`s assistant | Diclofenac on demand |
| 18, F | 61 | 3 | Technician | Lidocaine injections, tramadol |
| 19, F | 59 | 11 | Store clerk | None |
| 20, F | 61 | 50 | Office worker (retired) | Trimipramine |
| 21, F | 54 | 24 | Doctor´s assistant | None |
| 22, M | 50 | 16 | Administration secretary | Amitriptyline |
| 23, F | 70 | 36 | Master of home economy | Local anesthesia, metamizol |
| 24, F | 53 | 11 | Tailor | None |
| 25, F | 64 | 39 | Secretary | Ibuprofen on demand; homoeopathic substances |
| **Monopolar depression** |  |  |  |  |
| 1, M | 53 | 33 | Farmer | Amitriptyline |
| 2, F | 62 | 21 | Midwife (retired) | Venlafaxine |
| 3, F | 44 | 24 | Nurse | Quetiapine, tranylcypromine, aripiprazole, lithium |
| 4, F | 46 | 10 | Clerk | Venlafaxine, mirtazapine |
| 5, F | 42 | 13 | Child care worker | Amitriptyline, lithium |
| 6, F | 55 | 10 | Housewife (highschool degree) | Quetiapine, amitriptyline, nortriptyline, mirtazapine |
| 7, F | 42 | 22 | Clerk | Venlafaxine, risperidone |
| 8, F | 74 | 24 | Housewife (highschool degree) | Amitriptyline |
| 9, F | 55 | 3 | House cleaner | Venlafaxine |
| 10, F | 38 | 24 | Housewife (highschool degree) | Lorazepam |
