# Supplementary material for: Increased cortical activation upon painful stimulation in fibromyalgia syndrome
Source: BMC Neurol. 2015 Oct 20;15:210. doi: 10.1186/s12883-015-0472-4 (PMC4618366; doi:10.1186/s12883-015-0472-4)
Supplement: Additional file 2: Figure S1. — Flow-chart of patient enrolment. The flow-chart shows the process of eligibility testing of the FMS patients before enrolment. (PPT 72 kb) [file 12883_2015_472_MOESM2_ESM.ppt]

## Slide 1
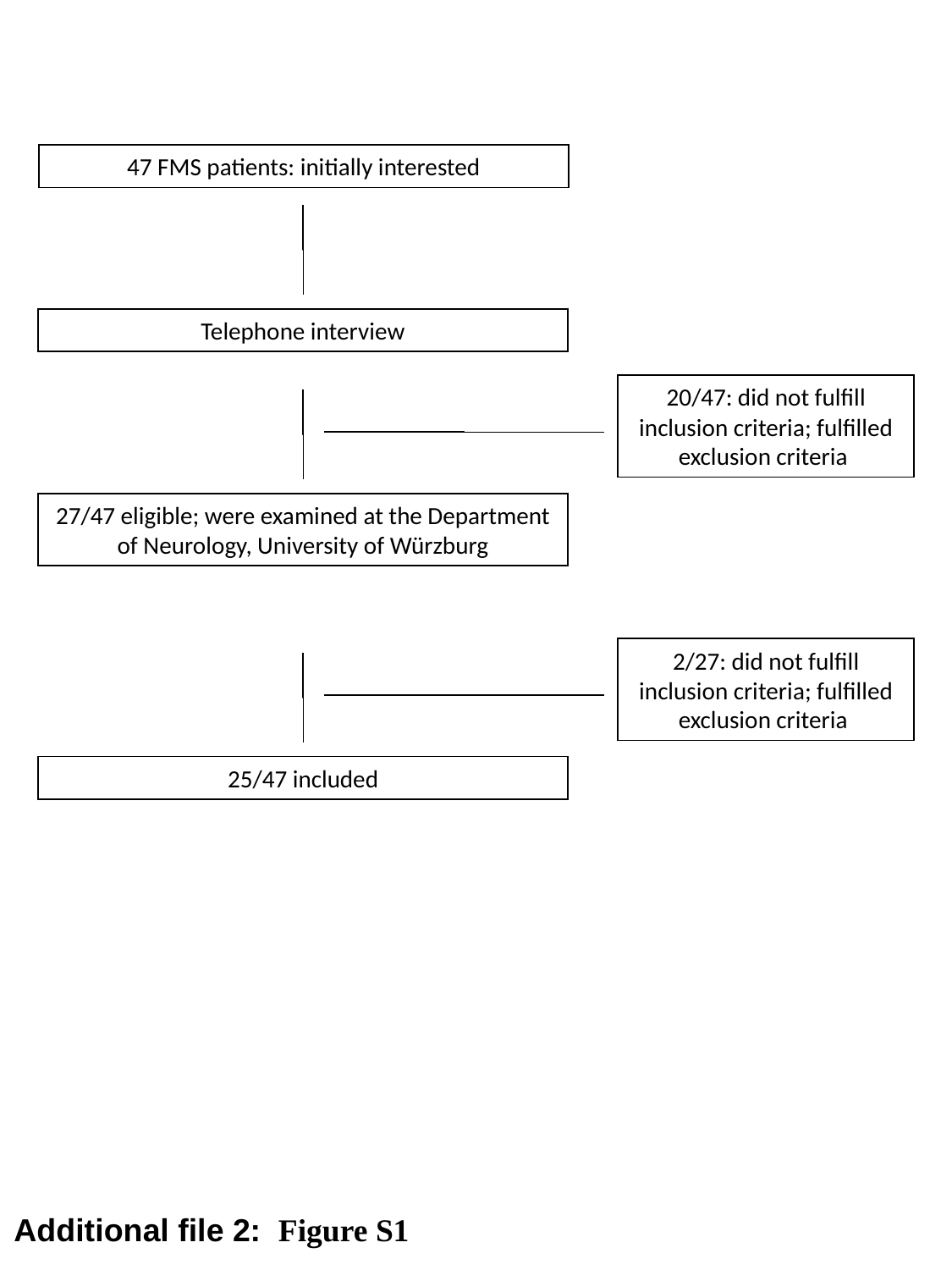

47 FMS patients: initially interested
Telephone interview
20/47: did not fulfill inclusion criteria; fulfilled exclusion criteria
27/47 eligible; were examined at the Department of Neurology, University of Würzburg
2/27: did not fulfill inclusion criteria; fulfilled exclusion criteria
25/47 included
Additional file 2: Figure S1
