# Supplementary figures and images for: Increased cortical activation upon painful stimulation in fibromyalgia syndrome
Source: BMC Neurol. 2015 Oct 20;15:210. doi: 10.1186/s12883-015-0472-4 (PMC4618366; doi:10.1186/s12883-015-0472-4)

## Slide 1
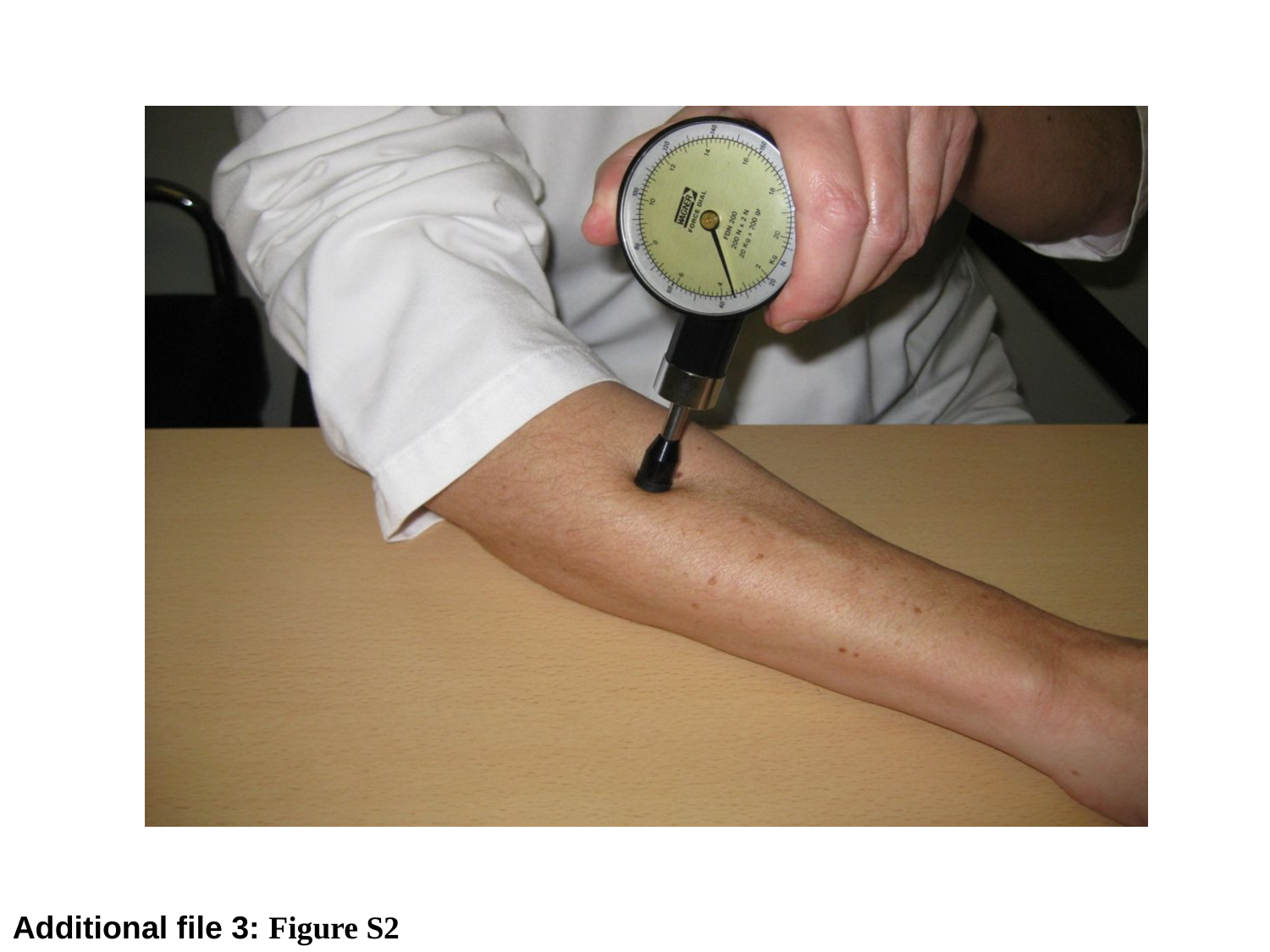

Additional file 3: Figure S2

Supplement: Additional file 3: Figure S2. — Pressure pain stimulation. A technician of our group demonstrates the application of pressure on the muscle bulk of the finger extensors using a calibrated algesiometer (Wagner Instruments, USA). (PPT 2368 kb) [file 12883_2015_472_MOESM3_ESM.ppt]

## Slide 1
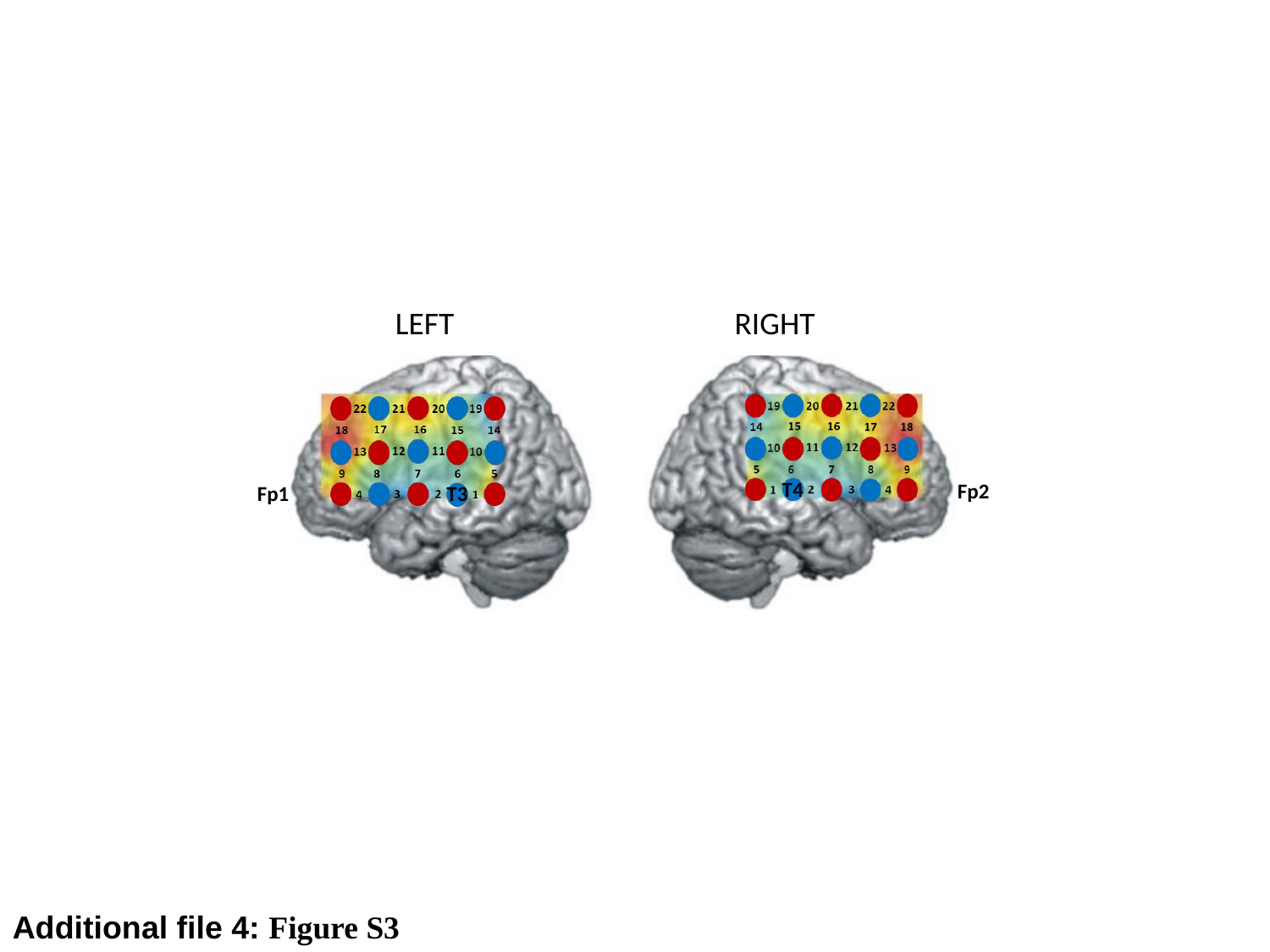

LEFT
RIGHT
T4
Fp2
Fp1
T3
Additional file 4: Figure S3

Supplement: Additional file 4: Figure S3. — Positioning of probe sets. The positions of the emitters (red dots) and the detectors (blue dots) are illustrated over the left and right hemisphere. The channels between the optodes are consecutively numbered. T3, T4, Fp1 and Fp2 are standard electroencephalography points that mark the positions of the detectors between channel 1 and 2 on the left side and channel 3 and 4 on the right side. (PPT 208 kb) [file 12883_2015_472_MOESM4_ESM.ppt]

## Slide 1
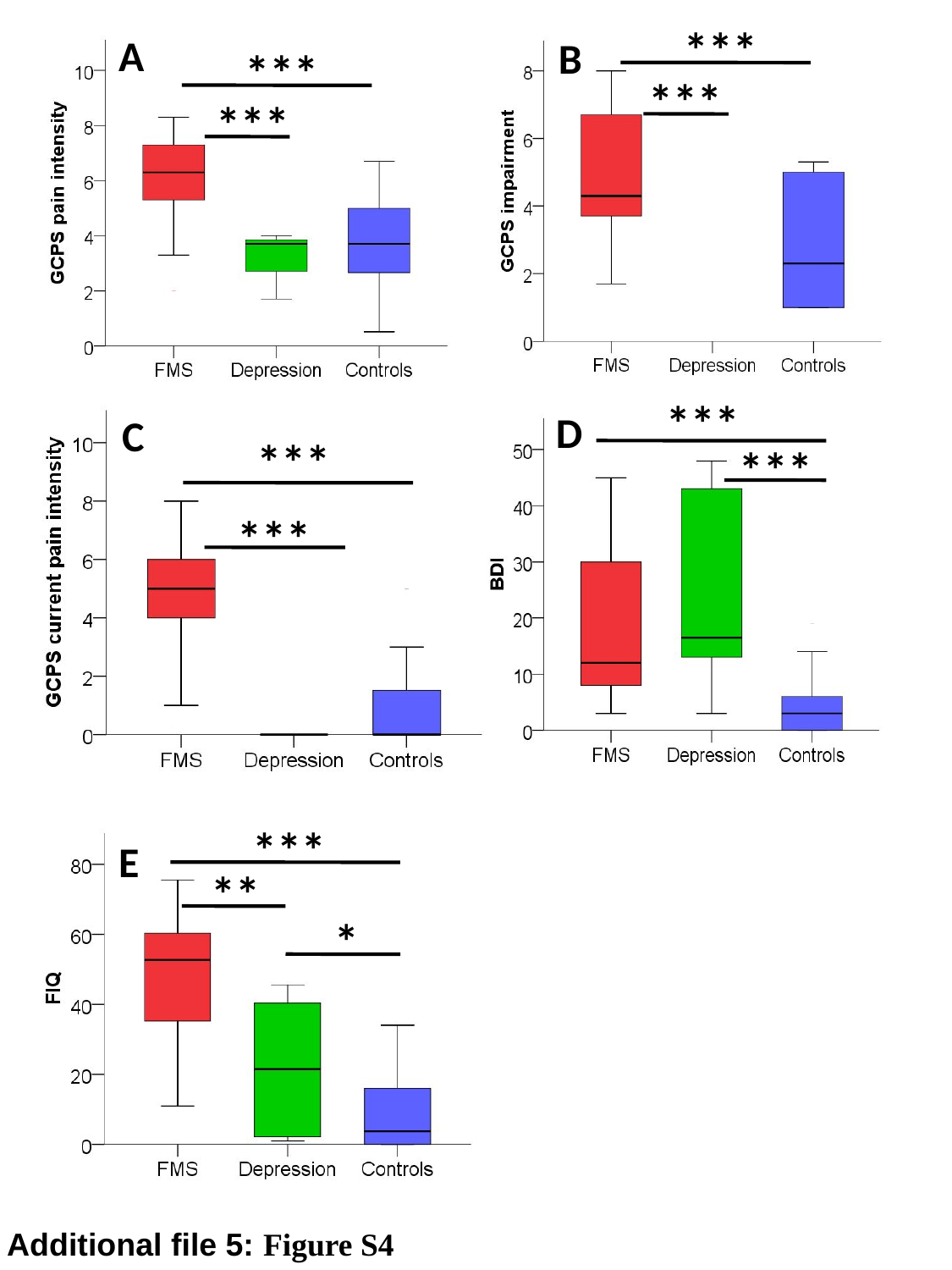

***
A
B
***
***
***
***
***
D
C
***
***
***
E
**
*
Additional file 5: Figure S4

Supplement: Additional file 5: Figure S4. — Results of questionnaires for pain, depression, and FMS symptoms. The boxplots give the results of the questionnaire assessment of the study participants. The median, and the first and third quartile are illustrated. (A) Patients with FMS had a higher median pain intensity in the last four weeks as measured by the Graded Chronic Pain Scale (GCPS) compared to patients with depression and to controls. (B) Also impairment of daily life due to pain in the last four weeks was greater in the FMS group compared to the other groups. (C) When asked for the current pain intensity FMS patients reported higher scores on a numerical rating scale (zero: no pain; ten: worst imaginable pain) then patients with depression or healthy controls. (D) In the Beck Depression Inventory (BDI) depressive symptoms were present in the FMS group and also in the group with unipolar depression compared to controls. (E) The Fibromyalgia Impact Questionnaire (FIQ) revealed higher scores for the FMS group compared to the depression group and the controls. *p < 0.05, **p < 0.01, ***p < 0.001. (PPT 107 kb) [file 12883_2015_472_MOESM5_ESM.ppt]

## Slide 1
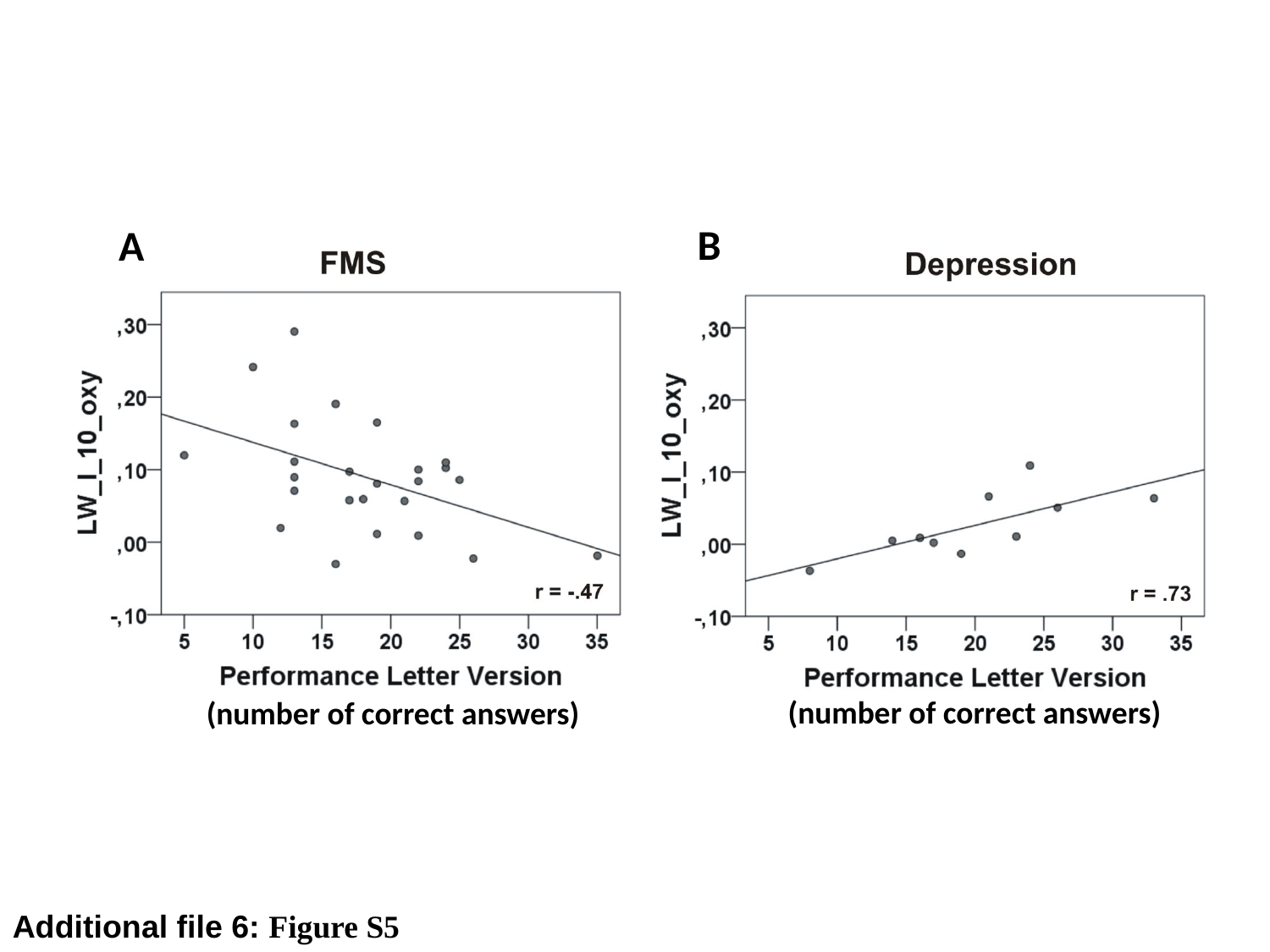

B
A
(number of correct answers)
(number of correct answers)
Additional file 6: Figure S5

Supplement: Additional file 6: Figure S5. — Correlation of performance in the verbal fluency test and cortical activation. Correlation between the task related activation in channel 10 over the left hemisphere and the number of correct answers during the letter version of the verbal fluency test (VFT) for patients with FMS and depression (p < 0.05). (PPT 323 kb) [file 12883_2015_472_MOESM6_ESM.ppt]
